# Mind the gap! the lack of concordance in diagnostic in liaison psychiatry in a portuguese hospital

**DOI:** 10.1192/j.eurpsy.2021.1735

**Published:** 2021-08-13

**Authors:** J. Marques, M. Silva, C. Laureano

**Affiliations:** 1 Psychiatry, Centro Hospitalar Universitário do Algarve, Portimão, Portugal; 2 Serviço De Psiquiatria, Centro Hospitalar de Leiria, Leiria, Portugal

**Keywords:** Concordance, liaison psychiatry, Diagnostic

## Abstract

**Introduction:**

Neurosciences evolved very rapidly in last few years and helped the establishment of Liaison Psychiatry as a fundamental part of the general hospitals functioning. However, the use of this department by the other specialties still needs to be refined, as it is common to find wrong assessments in the referral of the patients.

**Objectives:**

We aim to study the concordance between the referral motives and the assessment by the psychiatry team.

**Methods:**

Data was collected through the informatic registry. Contains patient data observed by a liaison psychiatrist in the period between 1^st^ of July and 30^th^ of September of 2020. In this period there were 80 requests, of which, 6 were refused for various reasons. We decided to study the concordance when one of these symptoms were in the request: anxious symptoms, depressive symptoms, psychotic symptoms and psychomotor agitation. 46 requests met this criteria.

**Results:**

The mean age was 63,3yo and 46% were older than 65yo. Most were women (54%) and 68% had history of psychiatry disorder. About 50% were requests from the Medicine wards. The concordance between the medical request and the psychiatry assessment was higher for psychomotor agitation (n=11; 64%) and depressive symptoms (n=23; 57%), but it was lower in anxious symptoms (n=3; 33%) and in psychotic symptoms (n=9; 33%). Most common diagnosis was delirium.

**Conclusions:**

Non-psychiatrist doctors appear to have more difficulty when assessing anxious and psychotic symptoms. Those concordance percentages are in line with recent research. Actions should be taken to improve this, like academic training and standardization of referral.

**Disclosure:**

No significant relationships.

